# Impact of Pelvic Calcification Severity on Renal Transplant Outcomes: A Prospective Single-Center Study

**DOI:** 10.3390/jcm13206171

**Published:** 2024-10-16

**Authors:** Iva Žuža, Doris Dodig, Ivan Brumini, Mate Kutlić, Robert Đurić, Nataša Katalinić, Antun Gršković, Ante Jakšić, Martina Mavrinac, Tanja Ćelić, Sanjin Rački, Lidija Orlić, Jasna Nekić, Dean Markić

**Affiliations:** 1 Department of Diagnostic and Interventional Radiology, Clinical Hospital Center Rijeka, 51000 Rijeka, Croatia; brumini92@gmail.com; 2 European Telemedicine Clinic, 08005 Barcelona, Spain; doris_5na5@yahoo.com; 3 Faculty of Medicine, University of Rijeka, 51000 Rijeka, Croatia; mate.kutlic@student.uniri.hr (M.K.); robert.djuric@student.uniri.hr (R.Đ.); natasa.katalinic@medri.uniri.hr (N.K.); antun.grskovic@medri.uniri.hr (A.G.); ante.jaksic@medri.uniri.hr (A.J.); tanja.celic@medri.uniri.hr (T.Ć.); sanjin.racki@medri.uniri.hr (S.R.); lidija.orlic@medri.uniri.hr (L.O.); dean.markic@medri.uniri.hr (D.M.); 4 Clinical Institute of Transfusion Medicine, Clinical Hospital Center Rijeka, 51000 Rijeka, Croatia; 5 Department of Urology, Clinical Hospital Center Rijeka, 51000 Rijeka, Croatia; 6 Faculty of Educational Sciences, University of Pula, 52100 Pula, Croatia; martina.mavrinac@unipu.hr; 7 Department of Nephrology, Dialysis and Transplantation, Clinical Hospital Center Rijeka, 51000 Rijeka, Croatia; 8 Department of Nuclear Medicine, Clinical Hospital Center Rijeka, 51000 Rijeka, Croatia; jasna.nekic@gmail.com

**Keywords:** kidney transplantation, kidney transplant outcomes, tomography, spiral computed, vascular calcification

## Abstract

**Background:** Vascular calcifications (VC) are increasingly prevalent in patients with chronic kidney disease. This study aimed to assess the incidence of iliac artery calcifications in kidney transplant (KT) patients and explore the relationship between iliac VC burden measured by pelvic calcification score (PCS) and renal transplant outcomes. **Methods:** This prospective study involved 79 KT recipients. VC quantification, using a pre-transplant computed tomography (CT) scan, was performed by assessing calcifications in the common and external iliac arteries bilaterally, resulting in an overall PCS ranging from 0 (no calcifications) to 44 (extensive calcifications). Based on PCS values, patients were divided into three equal-sized groups: PCS Group 1 (PCS 0–4), PCS Group 2 (PCS 5–19), and PCS Group 3 (PCS > 19). Post-transplant outcomes tracked for at least 1 year were patient and graft survival, graft function (urea, creatinine, MAG-3 clearance), and incidence of MACE during the first post-transplant year. **Results:** Calcifications were present in at least one arterial segment in 61 patients (77.2%). One-year patient survival was 95%, and one-year graft survival was 92.4%. Patients in PCS Group 3 had significantly lower one-year patient and graft survival compared to those in PCS Group 1 and 2 (*p* = 0.006 and *p* = 0.008, respectively). MACE and renal function indicators 1-year post-transplant were similar across all PCS groups. **Conclusions:** Our study demonstrated that a significant majority of KT recipients exhibited iliac VC during pre-transplant CT assessments. Patients in PCS Group 3 exhibited significantly lower one-year patient and graft survival rates compared to those in PCS Groups 1 and 2, indicating that this subgroup may require more intensive post-transplant monitoring and management.

## 1. Introduction

Vascular calcifications (VC) are commonly associated with aging, impaired kidney function, smoking, glucose resistance, dyslipidemia, hypertension, and disturbances in mineral and bone metabolism [1]. The relationship between VC, its associated risk factors, and subsequent clinical outcomes has been extensively studied [2]. Most research has concentrated on coronary artery calcifications, which have been strongly linked to cardiovascular events, particularly myocardial infarction [3,4]. Another significant area of investigation involves VC in the aortoiliac region [5], which is of particular importance for patients with end-stage kidney disease (ESKD) who are candidates for kidney transplantation (KT).

Patients with chronic kidney disease (CKD) exhibit a heightened incidence of arterial calcifications, driven by various factors such as advanced age, hyperparathyroidism, diabetes mellitus, and hypercholesterolemia [6,7,8]. Abdominal arterial calcifications have been linked to an increased risk of cardiovascular disease and mortality in both CKD patients and kidney transplant recipients [9,10]. From a surgical perspective, VC significantly influences the planning of kidney transplantation, including the selection of the implantation site and the choice of the appropriate arterial segment for anastomosis, typically the external iliac artery [11]. Furthermore, the relationship between VC and post-transplant outcomes, including patient and graft survival, has been the subject of recent investigations [11,12,13,14,15,16,17,18,19]. While some studies have identified a significant association between VC and adverse post-transplant outcomes, others have not [11,12,16,17,18,19].

The pre-transplant assessment of VC was traditionally conducted by pelvic X-rays and Doppler ultrasound of the iliac vessels. However, these methods have largely been supplanted by computed tomography (CT) [12,16,20]. In 2016, Davis introduced a novel CT-based scoring system designed to quantify iliac artery calcifications in the pre-transplant population [12]. This semiquantitative method evaluates the extent and distribution of calcifications based on morphological characteristics, degree of circumferential involvement, and the length of the affected arterial segments. Calcifications are assessed separately for each segment of the common and external iliac arteries. Building on the Davis scoring system, our group developed a refined CT-based scoring method called the pelvic calcification score (PCS), which quantifies the total burden of calcifications across both common and external iliac arteries [16]. In this prospective study, we investigated the correlation between pelvic calcification burden (measured by PCS and PCS groups) and renal transplant outcomes.

## 2. Materials and Methods

### 2.1. Patient Population and Clinical Outcomes

In this single-center, prospective study, we included 79 white patients who underwent KT at the Clinical Hospital Center Rijeka, Rijeka, Croatia, between 21 May 2021 and 31 August 2023.

Demographic data, including sex and age, along with clinical data such as body mass index (BMI), primary disease, comorbid conditions, type and duration of renal replacement therapy, transplantation site, arterial segment utilized for anastomosis, length of hospitalization, and graft and overall patient survival, were collected from the hospital’s medical records database. Comorbid conditions assessed at the time of transplantation included diabetes mellitus, arterial hypertension, hyperlipidemia, and hyperparathyroidism. The duration of renal replacement therapy was documented in months, and patients were classified according to the type of therapy received: hemodialysis, peritoneal dialysis, or preemptive kidney transplantation.

The donor origin was categorized as either from Croatia or other Eurotransplant countries. Major adverse cardiovascular events (MACE) were defined to include myocardial infarction, cerebrovascular event, cardiovascular death, unstable angina pectoris, peripheral arterial disease requiring revascularization (either endovascular or open surgery), heart failure necessitating hospitalization, and deep venous thrombosis. Patients were monitored for a minimum of one year following KT, with the longest follow-up period extending to 36 months. Regular assessments were conducted by both a nephrologist and a urologist. Notably, no patients were lost to follow-up at any point during the study. Post-transplant outcomes were assessed in terms of graft and patient survival. Additional outcomes included graft function one year after KT, evaluated by serum urea and creatinine levels as well as MAG-3 clearance via renal scintigraphy, and the incidence of MACE within the first year post-transplant.

### 2.2. Image Analysis

During the standard pre-transplant assessment, all patients underwent CT of the abdomen and pelvis to evaluate the iliac arteries, with a particular focus on detecting calcifications. All CT scans were performed within one year prior to KT on a Siemens Somatom Definition AS CT scanner (Siemens Medical Systems, Forchheim, Germany). Of the 79 patients, 57 underwent native, and the remaining 22 patients underwent post-contrast CT scans. Contrast-enhanced CT was performed when the surgeon (urologist) requested a more detailed evaluation of iliac artery anatomy or when there was a suspicion of severe stenosis based on significant vascular calcification identified on the native CT scan. Multiplanar reconstructions were reviewed using standard bone window settings (width 1500 HU, level 300 HU) by an experienced radiologist (I.Ž.) and senior radiology resident (I.B.) who were blinded to patient data and were experienced in assessing VC. The analysis was performed on SyngoVia workstation (Siemens Medical Solutions, Forchheim, Germany).

### 2.3. Vascular Calcification Measurement

Vascular calcifications were assessed using the semiquantitative scoring system proposed by Davis et al. [12]. This method evaluates three main calcification characteristics: morphology, circumference, and length. Morphology was categorized as follows: no calcification (score 0), thin linear calcification <1 mm in maximal thickness (score 1), linear calcification >1 mm in thickness (score 2), and bulky calcification >2 mm in thickness with convex luminal margins (score 3). The length and the degree of circumferential involvement of an arterial segment were graded as no calcification (score 0), 1–25% (score 1), 26–50% (score 2), 51–75% (score 3), and >76% (score 4) involvement.

VC was analyzed bilaterally in the common iliac artery (CIA) and external iliac artery (EIA). The CIA was anatomically defined from the inferior aspect of the aorta to its bifurcation, while the EIA extended from the CIA bifurcation to the level of the femoral head. The calcification score (CS) for each arterial segment was calculated as the sum of all calcification features, with possible scores ranging from 0 to 11. The overall pelvic calcification score (PCS) was then determined by summing the CS of all four arterial segments, with a total possible score ranging from 0 to 44. Based on the PCS, patients were stratified into three tertiles: PCS Group 1 (PCS 0 to 4), PCS Group 2 (PCS 5 to 19), and PCS Group 3 (PCS greater than 19).

### 2.4. Renal Transplant Technique and Immunosuppression Protocol

KT was performed in the iliac fossa using a standard previously reported surgical technique [21]. The kidney was preferably transplanted into the contralateral iliac fossa, with the primary objective of positioning the renal pelvis as the most superficial hilar structure. In cases of severe iliac artery calcifications, anatomical abnormalities, previously implanted grafts, or other contraindications for the KT in the contralateral iliac fossa, the kidney was implanted on the same side using the upside-down technique. This involved a 180-degree rotation of the kidney, such that the upper pole of the kidney became the lower pole. The external iliac arteries and veins were the preferred sites for renal anastomosis, while the common iliac arteries and veins were used when necessary.

The standard immunosuppression protocol for transplant recipients included induction therapy with basiliximab, followed by maintenance therapy with tacrolimus, mycophenolate mofetil, and prednisone.

### 2.5. Statistical Analysis

Statistical analysis was conducted using MedCalc version 20.021 (MedCalc Software, Mariakerke, Belgium). The normality of data distribution was assessed with the Kolmogorov–Smirnov test. Qualitative variables, such as primary disease, gender, and comorbid conditions, were presented as absolute and relative frequencies. Quantitative variables, including patient and graft survival, age, BMI, duration of hospitalization, and length of dialysis, were reported as medians along with the 5th and 95th percentiles. Inter-rater agreement was calculated using the intraclass correlation coefficient (ICC), and the values were determined as follows: >0.50, poor; 0.50 to 0.75, moderate; 0.75 to 0.90, good; and >0.90 excellent agreement [22]. The frequency of analyzed variables was compared using the Chi-square test and post hoc test of proportion. Differences between PCS groups were calculated using Mann–Whitney or Kruskall–Wallis tests. Post-hoc analysis was performed with the Dunn test when multiple comparisons were made. The involvement of iliac arteries with VC, depending on morphology, circumference, and length of the arterial segment, was calculated using a Chi-square test and a *t*-test of proportion as a post hoc test. The survival of grafts and patients was determined by Kaplan–Meier survival analysis and the log-rank test. Spearman’s rank correlation coefficient was used to calculate the correlation between PCS and age, duration of dialysis, duration of operation, length of hospitalization, graft survival, patient survival, and MACE.

All statistical values were considered significant at a *p*-level below 0.05.

### 2.6. Ethical Approval

The study was conducted according to the guidelines of the Declaration of Helsinki and approved by the Ethical Committee of Clinical Hospital Center Rijeka, Rijeka, Croatia (protocol code: 003-05/21-1/57/2021), date of approval 13 May 2021. Informed consent was obtained from all subjects involved in the study.

The study was registered on the ClinicalTrials platform (Clinical Hospital Center Rijeka Protocol Record 3105, Vascular Calcifications in Kidney Transplant Recipient: NCT05697458).

## 3. Results

### 3.1. Patient Characteristics and PCS

The study included 79 patients who underwent kidney KT at our institution. The median age of the cohort was 59 years (range: 16 to 76), with male predominance (63.3%). The median BMI was 26 kg/m^2^ (range: 16.65 to 43.71). The median duration of dialysis prior to KT was 24 months (range: 0 to 132), the median cold ischemia time was 910 min (range: 120 to 1660), and the median duration of surgery was 180 min (range: 105 to 290). (see Table 1). Parathyroid hormone was elevated in KT patients (Table 2).

The most common causes of ESKD were chronic glomerulonephritis, nephroangiosclerosis, polycystic kidney disease, and diabetes mellitus. Other causes of ESKD included bilateral congenital renal hypoplasia, staghorn renal stones, Wegener granulomatosis, hemolytic uremic syndrome, neurogenic bladder, tetralogy of Fallot, endemic nephropathy, obstructive uropathy, thrombotic thrombocytopenic purpura, and vesicoureteral reflux. For eight patients, this was their second KT.

Ipsilateral KT was performed in 11 patients (13.9%). The reasons for choosing ipsilateral KT included extensive calcifications of the iliac arteries on the contralateral side, as revealed by preoperative CT scans (three patients); enlarged polycystic kidneys (two patients); a preexisting graft (two patients); hypotrophic contralateral external iliac artery (two patients); a prior operation on the contralateral iliac artery following an iatrogenic injury during a renal denervation procedure (one patient); and urinary augmentation with the colon (one patient). Forty-six percent of patients were without early complications, and 26% had some urological, vascular, or infective complications. Other early complications (28%) included delayed graft function (4 patients), anemia with the need for blood transfusion (13 patients), lymphocele (3 patients), laparocele (1 patient) and subcutaneous bleeding (1 patient).

Late complications were observed in four patients (5.1%). These included a retroperitoneal abscess occurring 12 months post-transplant, hydronephrosis caused by a ureteral stone also after 12 months, bacterial endocarditis with valvular vegetations and septic emboli at 6 months, and stenosis of the ureterovesical anastomosis at 6 months. With the exception of bacterial endocarditis, all late complications were managed surgically.

MACE within the first post-transplant year was diagnosed in 15 patients (18.9%). The most common was unstable angina pectoris (five patients), followed by peripheral arterial disease requiring revascularization procedures (four patients), cardiac decompensation necessitating hospitalization (two patients), deep venous thrombosis (two patients), cerebrovascular accident (one patient), and myocardial infarction (one patient).

During the first post-transplant year, six grafts were lost. The causes of graft loss included a pseudoaneurysm of the external iliac artery (one patient), a urinary fistula with an iatrogenic injury to the renal artery during pyeloureterostomy (one patient), and four patient deaths with functioning grafts (sepsis in one patient and sudden death at home in three patients). During the first post-transplant year, four patients died, all with a functioning graft. The demographic and clinical characteristics of the patients, including their PCS, are presented in Supplementary File, Table S1.

### 3.2. Calcification Analysis

**Figure 1:**
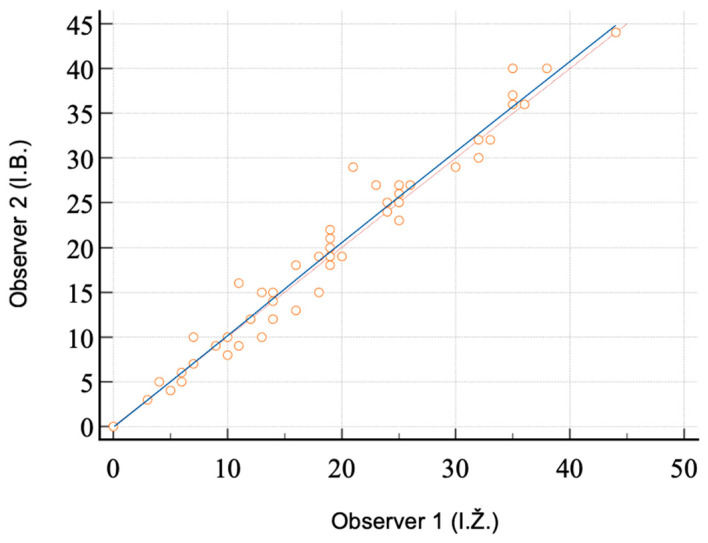
Relationship between two observers (I.Ž. and I.B.) in the assessment of calcifications of iliac arteries (scatter diagram).

The inter-rater correlation between two radiologists for an assessment of arterial calcifications for all arterial segments was excellent (r = 0.989 with 95% CI 0.984 to 0.993), and the dataset from one reader (I.Ž.) was used for further statistical analysis (see Figure 1).

The median PCS value for the entire cohort was 12 (range 0–44). PCS was significantly higher in patients older than 55 years (*p* < 0.001) and in patients with hypertension, diabetes, and hyperlipidemia (*p* = 0.017, *p* < 0.001, *p* < 0.001, respectively). The PCS value was higher in patients with diabetes and nephroangiosclerosis as the principal disease. In three patients in which extensive calcifications of the external iliac arteries precluded contralateral KT, the PCS values were 25, 38, and 44.

### 3.3. Iliac Artery Calcification Distribution

Sixty-one patients (61/79, 77.2%) had calcification on at least one analyzed iliac artery. Similarly, calcification morphology, circumference, and length scores were higher in common iliac arteries and on the right side (see Table 3).

The frequency of calcification differed significantly across the four arterial segments (*p* < 0.001), with the highest prevalence observed in the common iliac arteries (*p* < 0.001). (see Table 4).

Post-hoc analysis showed a significant difference between the right CIA and both the right and the left EIA (all *p* < 0.001). Also, there was a significant difference between the left CIA and both the right and the left EIA (respectively, *p* = 0.005 and *p* < 0.001). Calcification frequency did not show a significant difference between the right and left sides for both CIA and EIA (*p* = 0.307 and *p* = 0.714, respectively).

### 3.4. Pelvic Calcification Score Groups

Patients were divided into three groups based on their individual PCS, as shown in Table 5. Group 1 included patients with PCS below 5, Group 2 comprised those with PCS between 5 and 19, and Group 3 consisted of patients with PCS above 19.

Table 6 presents VC in each arterial segment across all three PCS groups. The frequency of calcifications was significantly higher in PCS Group 3 for both the right and left EIA (all *p* < 0.001). Additionally, the VC frequency in the right CIA was statistically higher in PCS Group 3 (*p* < 0.001). The VC levels of the left CIA were significantly higher in both PCS Groups 2 and 3 compared to PCS Group 1 (*p* < 0.001).

Using Spearman’s rank correlation coefficient, we identified a statistically significant positive correlation between pelvic calcification score and patient age (refer to Supplementary File, Table S2).

### 3.5. PCS Groups and Patient Characteristics

Patients in Group 1 were significantly younger than those in Groups 2 and 3 (*p* < 0.05) (see Table 7). Although most patients with diabetes and MACE were in Group 3, the difference was not statistically significant.

### 3.6. Correlation of PCS Groups with Post-Transplant Renal Function and MACE

Renal function indicators one year after KT are comparable across all three PCS groups (Table 8). Although the number of patients with MACE is higher in PCS Group 3, the difference is not statistically significant (see Table 7).

### 3.7. Overall Graft and Patient Survival across PCS Groups

**Figure 2:**
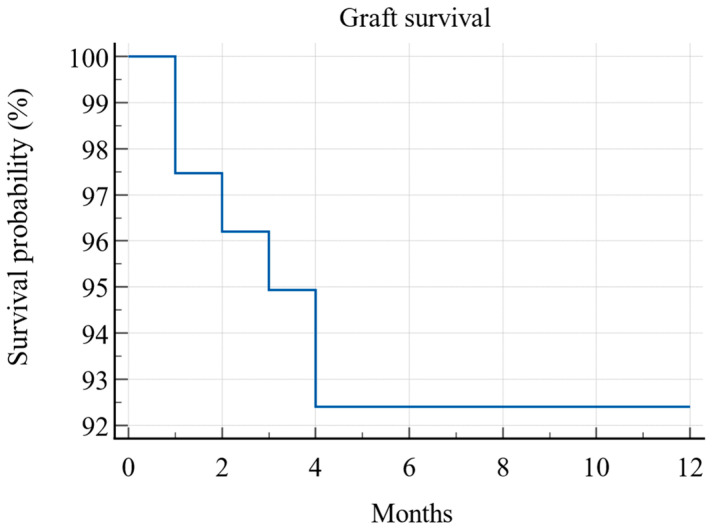
Kaplan–Meier survival curve of overall graft survival during the first post-transplant year (N = 79).

**Figure 3:**
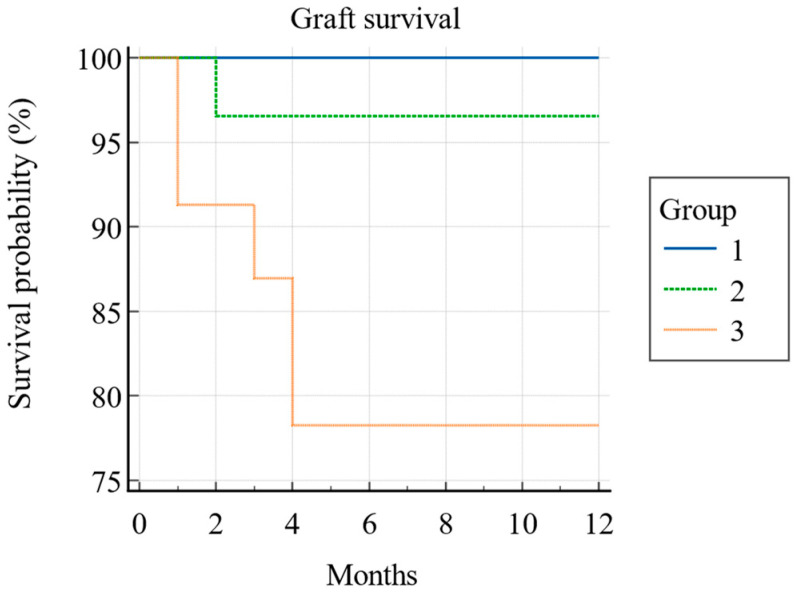
Kaplan–Meier survival curve of overall graft survival between three PCS groups during the first post-transplant year. (N = 79).

During the first post-transplant year, six grafts (7.6%) were lost, resulting in an overall one-year graft survival rate of 92.4% (Kaplan–Meier) (see Figure 2). Of these, five were from PCS Group 3, and one was from PCS Group 2. The one-year graft survival rate was 100% for Group 1, 97% for Group 2, and 78% for Group 3, with the difference being statistically significant (*p* = 0.008; log-rank test) (see Figure 3).

**Figure 4:**
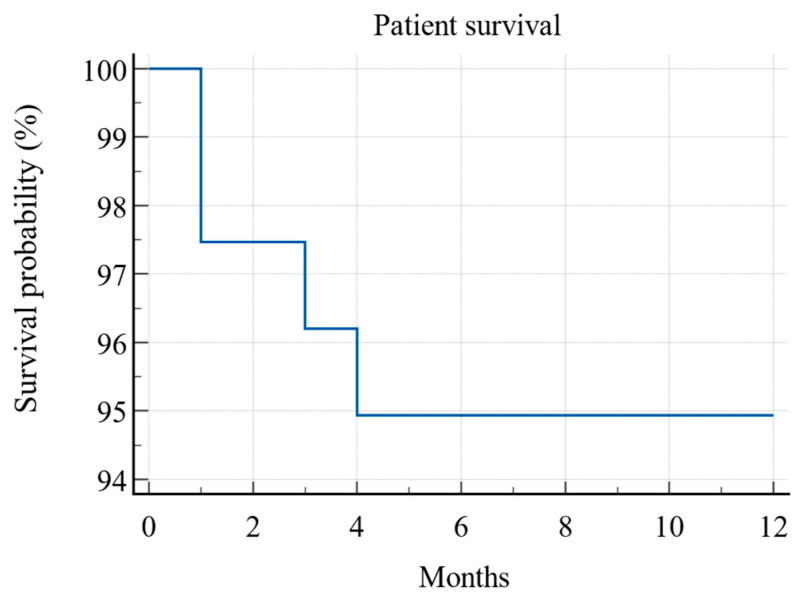
Kaplan–Meier survival curve of patient survival during the first post-transplant year (N = 79).

**Figure 5:**
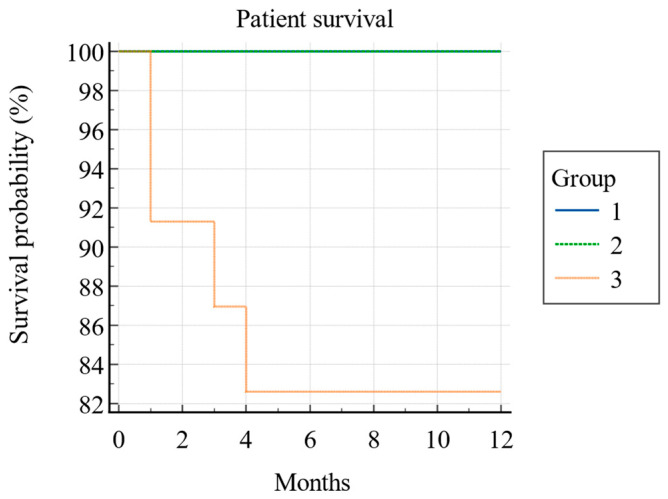
Kaplan–Meier survival curve of patient survival between three PCS groups during the first post-transplant year (N = 79).

In the first year after KT, four (5%) patients died, leading to a one-year patient survival rate of 95% (Kaplan–Meier) (see Figure 4). All the deceased patients were from PCS Group 3, and this difference was also statistically significant (*p* = 0.006; log-rank test) (see Figure 5).

## 4. Discussion

The pre-transplant evaluation of potential kidney recipients typically includes an assessment of the iliac vessels. While CT is considered the most accurate method for detecting calcifications in the iliac arteries, some international organizations, such as the European Association of Urology, Kidney Disease Improving Global Outcomes, and the European Renal Association, do not currently recommend routine CT evaluation in their guidelines [20,23,24,25]. Since the iliac arteries are commonly used for vascular anastomosis in KT, extensive calcifications in these vessels may influence the surgical approach and, in some cases, even make it impossible.

The right and left EIA were more frequently used for KT than the right and left CIA (*p* < 0.001), which is consistent with standard surgical practice, where EIAs are predominantly chosen for arterial anastomosis. As defined by the PCS group classification, patients in PCS Group 3 exhibited a higher burden of calcifications, including those affecting the EIAs, compared to patients in PCS Groups 1 and 2.

Few studies have evaluated the impact of pre-transplant assessment of iliac artery calcifications. In a retrospective analysis of 187 patients on the renal transplant waiting list, pelvic X-rays revealed that 39.5% of patients had moderate or severe calcifications, prompting further evaluation with CT angiography [11]. Following CT angiography, 21.9% of patients were removed from the waiting list due to extensive calcifications involving all iliac arteries. Additionally, CT findings influenced the selection of the transplantation site in 24.3% of the patients. Among the 22 patients who experienced vascular complications, 19 (86.4%) had moderate to extensive vascular calcifications. The authors concluded that pelvic X-ray is a valuable screening tool for identifying patients who may require more comprehensive vascular diagnostic evaluation prior to KT [11]. Aalten et al. conducted a prospective study to assess the utility of pelvic X-rays in identifying vascular calcifications and predicting arterial complications in patients undergoing KT. Preoperatively, VC was detected in 33 patients (30.2%) via pelvic X-ray and in 35 patients (32.1%) intraoperatively through surgical palpation. The sensitivity and specificity of the pelvic X-ray for detecting vascular calcifications in the iliac arteries were 48% and 82%, respectively. Arterial anastomosis complications attributable to calcifications were encountered in five patients (4.6%). The negative predictive value and positive predictive value of the pelvic X-ray for predicting complications with arterial anastomosis were 99% and 14%, respectively. These findings suggest that while the pelvic X-ray is not a reliable method for detecting vascular calcifications, the likelihood of technical complications with arterial anastomosis remains low in the absence of radiographic evidence of calcifications [17].

Contrary to earlier studies where preoperative vascular assessments were predominantly performed by X-ray, CT has become increasingly integral to the pre-transplant evaluation process. Consistent with the higher sensitivity of CT in detecting vascular calcifications compared to radiography, calcifications were identified in at least one segment of the iliac arteries in 77.2% of our patients, closely aligning with the 69% reported in the study by Davis et al. [12]. Additionally, similar to findings from other studies, we observed that the CIAs exhibited a greater prevalence of vascular calcifications compared to the EIAs bilaterally [12,13,16]. Furthermore, in a separate study involving 114 patients, severe calcifications detected by CT resulted in the exclusion of 33 patients (29%) from KT [20]. However, CT imaging was only conducted in a subset of patients with a higher incidence of VC, including those over 55 years of age, diabetic patients, individuals undergoing second transplants, and those with evident VC on plain abdominal X-rays [20]. Moreover, severe calcifications of the EIAs identified on CT were associated with increased surgical complexity and a higher incidence of post-transplant lower extremity amputation [13]. Consequently, the authors concluded that patients with significant EIA calcifications detected on preoperative CT scans should be referred to a vascular surgeon for further evaluation [13]. The aforementioned studies demonstrate that CT is a reliable and valuable method for evaluating calcifications in the iliac arteries of patients undergoing preparation for kidney transplantation [12,13,16,20].

One of the most valuable applications of CT in the kidney transplant population is the determination of the optimal implantation site and arterial segment for anastomosing the renal artery of the graft. In our cohort, CT imaging was crucial in determining the appropriate kidney transplant site in five patients (6.3%), where ipsilateral KT was ultimately performed. In three cases, the decision for ipsilateral KT was made due to severe calcifications of the contralateral iliac arteries, while in the remaining two patients, the contralateral iliac artery was deemed gracile. While some transplant centers may prefer performing KT on the right side due to the superficial position of the iliac vessels, we assert that the final decision regarding the implantation site should be guided by imaging findings, particularly those obtained from CT.

If the side and site are not adequately assessed preoperatively, the patient may require two incisions to identify a suitable arterial segment for anastomosis. Alternatively, a median extraperitoneal incision of the anterior abdominal wall could be performed, allowing exploration of both iliac fossae to locate an appropriate site for arterial anastomosis. Therefore, the use of CT during pre-transplant evaluation is invaluable for accurately determining the optimal side and site for anastomosis, thereby minimizing the need for additional incisions and reducing surgical complexity.

Quantification of iliac artery calcifications on CT has been utilized in only a few studies [12,13,16]. In this study, as well as in our previous research, we employed the PCS for the assessment and quantification of VC [16]. The excellent correlation between observers (r = 0.989, 95% CI 0.984 to 0.993) underscores the reliability and reproducibility of this method. To quantify arterial calcifications, we have adopted PCS as a semiquantitative scoring system [16]. PCS values range from 0, indicating no calcifications in any of the four arterial segments, to 44, indicating extensive calcifications in all arterial segments. This approach represents a significant advancement over studies that rely solely on descriptive assessments of calcifications [19,20,26].

Among the traditional risk factors for the development of VC, age is particularly notable. In our study, the median age was 59 years, which is consistent with the values reported in previous studies, with the exception of the Davis study, where the median age was 52 years [11,12,13,16,20]. Similar to other research, we demonstrated that iliac artery calcifications increase with age [11,12,16,26]. In our study, patients older than 55 years, as well as those with hypertension, diabetes, and hyperlipidemia, exhibited significantly higher PCS. Likewise, patients in Group 1 were younger than those in Groups 2 and 3, and patients in Group 2 were younger than those in Group 3.

The correlation between the iliac VC burden and patient or graft survival has not been clearly established [11,12,13,14,15,16,17,18,19]. A study involving 109 patients found no significant difference in 1-year graft and patient survival rates between those with palpable vascular calcifications in the iliac arteries and those without (94% vs. 97% for graft survival and 97% vs. 98% for patient survival) [17]. Additionally, no significant difference in post-transplant renal function was observed between patients with and without palpable vascular calcifications [17].

Disthabanchong et al. conducted an evaluation of vascular calcifications in the abdominal aorta and iliac arteries using plain radiographs in a cohort of 419 patients. This cohort included individuals with non-dialysis chronic kidney disease, those undergoing maintenance hemodialysis, and long-term kidney transplant recipients. The patients were followed prospectively over a period of 5 years. The study concluded that pelvic arterial calcifications serve as an independent predictor of mortality across all three patient groups [19]. In a separate study, Davis et al. retrospectively analyzed iliac artery calcifications using CT scans in 131 patients who underwent pelvic CT within two years prior to kidney transplantation [12]. Patients without iliac VC exhibited a significantly higher 3-year survival rate compared to those with any degree of iliac calcifications (94% vs. 87%). However, the calcification scores for the iliac artery segment used in the anastomosis did not show a significant correlation with either graft or patient survival. Notably, only the calcification morphology score of the arterial segment used for anastomosis was significantly predictive of increased surgical complexity and delayed graft function [12]. Werlin et al. conducted a retrospective analysis of 204 patients who underwent CT imaging within 3 years prior to kidney transplantation. The study utilized the dichotomized Davis classification to assess arterial calcifications, focusing exclusively on the arterial segments used for renal artery anastomosis. The findings revealed no significant differences in graft or patient survival when stratified according to any calcification score [13]. In another retrospective study involving 100 patients, where CT was used as the diagnostic tool, the aortoiliac calcification score was not associated with graft or patient survival [18]. However, in our previous research, patients with a PCS greater than 3 demonstrated significantly shorter graft and patient survival [16]. In a study involving 547 transplant patients, Benjamens utilized the aorto-iliac calcium score from CT scans to assess vascular calcifications, categorizing patients into low, medium, and high calcium score groups. The study found that patients with higher aorto-iliac scores had lower overall survival and experienced a greater number of cardiovascular events [14]. In our study, both graft and patient survival were better in those with lower PCS values. Notably, all patients who died within the first year post-transplant belonged to PCS Group 3, with a median PCS of 25, compared to a median PCS of 11 in survivors.

In our previous study, a PCS value of 3 was identified as the critical threshold for survival; specifically, patients with a PCS above 3 demonstrated shorter graft and patient survival [16]. It is clear that patients with a PCS of 3 or below either had no calcifications or only minimal calcifications. As anticipated, similar to findings in other studies, these patients exhibited a better prognosis compared to those with more significant vascular calcifications [12]. To enhance the precision of our PCS assessment, we stratified patients into tertiles of equal size. Given the range of calcifications from 0 to 44, it is statistically more appropriate to utilize a tertile distribution rather than relying on a single cut-off value. This approach ensures that patients with minimal calcifications (e.g., PCS of 3 or 4) are not grouped together with those having significantly higher PCS values (e.g., PCS of 15 or 20) in statistical analyses. One key finding from this tertile division is that patients in PCS Group 3 (PCS above 19, representing those with the most extensive calcifications) had significantly shorter graft and patient survival compared to those in PCS Group 1 (PCS 0–4) and Group 2 (PCS 5–19). Although we observed a difference in graft survival between Group 1 and Group 2, this difference was not statistically significant. Relying on a single cut-off value would lead to a less nuanced and less precise interpretation of the results. Our findings demonstrate that arterial calcifications are correlated with both graft and patient survival, suggesting that PCS groups could serve as valuable prognostic factors in kidney transplant patients.

In our study, kidney function one-year post-kidney transplantation was assessed using serum urea and creatinine levels, along with renal scintigraphy. No significant differences in kidney function were observed between the different PCS groups. This finding aligns with the study by Benjamens et al., which also reported no significant difference in estimated glomerular filtration rate (eGFR) across three groups with varying degrees of aorto-iliac artery calcifications. However, their study noted a non-statistically significant trend of lower eGFR as the calcium score increased [14]. In a similar study, inverse associations between pre-transplant aorto-iliac calcifications and one-year eGFR were identified in univariate analysis. However, these associations were not significant after adjusting for potential confounders, such as donor and recipient age [15]. In another study that used quartiles of aorto-iliac calcifications to quantify VC one year after KT, no significant differences in eGFR were observed among the groups. However, patients with higher levels of calcification tended to have lower eGFR values [18]. In a cohort of 93 transplant recipients stratified into three groups based on the degree of VC, there was no significant difference in one-year creatinine levels. The no/minimal VC group had an average creatinine value of 140.3 µmol/L, compared to 148.5 µmol/L in the moderate/severe VC group [11]. Rijkse et al., in a prospective study using non-contrast CT, investigated the association between aorto-iliac calcification (assessed by a modified Agatston score) on the side of transplantation and eGFR. The study found that aorto-iliac calcifications, measured in the aorta and common iliac artery, were not directly associated with eGFR during the first year post-transplant. However, patients with severe vascular calcification (VC) exhibited a lower eGFR trajectory [27].

The primary role of CT-based assessment of VC in the iliac arteries is for preoperative planning. However, it may also play a valuable role in the management and risk stratification of cardiovascular diseases, with the primary goal of reducing the incidence of MACE. Cardiovascular disease is particularly significant in the CKD population, as mortality from cardiovascular disease is 10 to 20 times higher in patients with ESKD compared to the general population [28]. In a separate retrospective study utilizing the dichotomized Davis classification, a median follow-up of 3.3 years revealed that 10% of patients experienced post-operative MACE, defined as myocardial infarction, coronary intervention, or cerebrovascular accident, following kidney transplantation [13]. They identified a correlation between MACE and VC in the common iliac arteries, particularly focusing on plaque morphology and the length of calcifications. Their study suggests that plaque burden in the common iliac arteries is associated with MACE and likely reflects a greater severity of systemic atherosclerotic disease [13]. In a dual-cohort study from the Netherlands, with a median follow-up of 3.1 years, cardiovascular events were observed in 15.5% of patients. Multivariable analysis in this study showed that the aorto-iliac calcium score was significantly associated with both cardiovascular mortality and cardiovascular events [14]. In our study, 19% of patients experienced MACE during the first post-transplant year. One possible reason for the higher incidence of cardiovascular events in our cohort could be our broader definition of MACE. We did not find a correlation between MACE and the PCS or PCS groups. Furthermore, no consistent patterns were observed regarding the type or timing of MACE following KT.

Additionally, three of our patients died suddenly at home, and it is possible that MACE contributed to their deaths. These findings support the notion that arterial calcifications in kidney transplant patients are associated with increased rates of post-transplant cardiovascular morbidity and mortality [9].

Our study has several limitations. Firstly, this study is limited by its single-center design, relatively small patient cohort, and short duration of follow-up. We acknowledge that a multi-center approach with an extended follow-up period would likely enhance statistical power, increase patient diversity, and provide a more comprehensive assessment of long-term outcomes. However, implementing such improvements may present logistical and practical challenges. Although we divided patients with VC into three groups using PCS tertiles to ensure more balanced group sizes, tertiles are inherently based on equal-size groups, making the results population-dependent. Therefore, external validation of these PCS groups is necessary to confirm our findings. Additionally, other potential confounding factors, such as medication adherence and lifestyle behaviors, may have influenced transplant outcomes but were not comprehensively analyzed in this study. Tacrolimus levels were regularly monitored, and in selected cases, mycophenolate mofetil levels, offering only a limited assessment of patients’ medication adherence.

The strengths of this study include its prospective design and the novel exploration of a possible correlation between PCS groups and transplant outcomes. Unlike other studies where CT was performed 2 or 3 years before KT, our study conducted CT assessments 1 year prior to KT. This timing allows for a more accurate measurement of calcification extent, given that VC progresses with age. Additionally, the quantification of VC using PCS and PCS groups is both reproducible and reliable, as demonstrated by a very high inter-reader correlation (ICC, r = 0.989, 95% CI: 0.984 to 0.993). Another key strength of our study is its broad representation of the entire transplant population, as we did not selectively exclude patients from CT assessment based on factors such as age, dialysis duration, smoking history, comorbidities, or ethnicity, unlike some other studies [27].

## 5. Conclusions

In conclusion, this prospective study demonstrates that CT-based quantification of iliac artery vascular calcifications is a valuable tool for predicting both graft and patient survival. Dividing patients into three groups based on their PCS values provides more precise prognostic information than using a single cut-off value. These findings could aid in identifying patients with potentially lower graft and overall survival, who may benefit from more stringent post-transplant follow-up to improve their prognosis after kidney transplantation.

## Figures and Tables

**Table 1:** Patient clinical data.

	N	Median	5th–95th Percentiles
Body mass index (kg/m^2^)	79	26	19–33
Duration of dialysis (months)	79	24	0–88
HLA matching	79	3	1–4.55
Cold ischemia time (minutes)	79	910	222–1490
Duration of operation (minutes)	79	180	115–235
Length of hospitalization (days)	79	14	9–40.55

**Table 2:** Values of parathyroid hormone (PTH), alkaline phosphatase, calcium, and phosphorus before kidney transplantation.

	N	Median	5th–95th Percentiles	Referral Values
Calcium (mmol/L)	79	2.3	2–2.7	2.14–2.53
Phosphorus (mmol/L)	79	0.87	0.43–2.35	0.79–1.42
Alkaline phosphatase (IU/L)	79	78	38–165	60–142
PTH (pmol/L)	79	16.79	6.26–54	1.6–6.9

**Table 3:** Frequency of calcifications in each arterial segment, categorized by morphology, circumference, and length.

	ARTERIAL SEGMENT			
	Right Common Iliac Artery	Left Common Iliac Artery	Right External Iliac Artery	Left External Iliac Artery
	N (%)			
Morphology				
0	20 (25.3)	27 (34.2)	54 (68.4)	59 (74.7)
1	12 (15.2)	10 (12.7)	8 (10.1)	9 (11.4)
2	6 (7.6)	10 (12.7)	4 (5.1)	1 (1.3)
3	41 (51.9)	32 (40.5)	13 (16.5)	10 (12.7)
*p*	<0.001	<0.001	<0.001	<0.001
Circumference				
0	20 (25.3)	27 (34.2)	54 (68.4)	58 (73.4)
1	25 (31.6)	19 (24.1)	13 (16.5)	12 (15.2)
2	11 (13.9)	12 (15.2)	7 (8.9)	5 (6.3)
3	13 (16.5)	12 (15.2)	3 (3.8)	3 (3.8)
4	10 (12.7)	9 (11.4)	2 (2.5)	1 (1.3)
*p*	<0.001	<0.001	<0.001	<0.001
Length				
0	20 (25.3)	27 (34.2)	54 (68.4)	58 (73.4)
1	19 (24.1)	17 (21.5)	15 (19.0)	14 (17.7)
2	5 (6.3)	5 (6.3)	5 (6.3)	2 (2.5)
3	4 (5.1)	5 (6.3)	3 (3.8)	1 (1.3)
4	31 (39.2)	25 (31.6)	2 (2.5)	4 (5.1)
*p*	<0.001	<0.001	<0.001	<0.001
Overall calcifications	59 (74.7)	52 (65.8)	25 (31.6)	21 (26.6)

**Table 4:** Calcification frequency in analyzed pelvic arteries (N = 79).

Arterial Segment	Calcification Frequency N (%)	*p*
Right common iliac artery	59 (74.7)	<0.001
Left common iliac artery	52 (65.8)	
Right external iliac artery	25 (31.6)	
Left external iliac artery	21 (26.6)	

**Table 5:** Formation of three patient groups based on their individual pelvic calcification score (N = 79).

	Groups and Division Criteria		
Group	1	2	3
Percentiles	0–33	33–66	66–100
Pelvic calcification score	0–4	5–19	>19
N (%)	27 (34.2)	29 (36.7)	23 (29.1)
*p*	0.702		

**Table 6:** Incidence of vascular calcifications in each arterial segment across pelvic calcification score (PCS) groups N = 79).

Arterial Segment	PCS Groups			*p*
	1 (N = 27)	2 (N = 29)	3 (N = 23)	
Presence of Arterial Calcifications	N (%)			
Right common iliac artery	8 (30)	28 (97)	23 (100)	<0.001
Left common iliac artery	1 (3.7)	28 (97)	23 (100)	<0.001
Right external iliac artery	0	5 (17)	20 (87)	<0.001
Left external iliac artery	0	3 (10)	18 (78)	<0.001

**Table 7:** Demographic and clinical patient data across three pelvic calcification score (PCS) groups.

Variables	PCS Groups			*p*
	1 (N = 27)	2 (N = 29)	3 (N = 23)	
	Median			
Age (years)	46 *	60 *	67 *	<0.001 *
Body mass index (kg/m^2^)	24.7	25.5	28.6	0.067
Duration of dialysis (months)	24	24	24	0.893
Cold ischemia time (minutes)	940	950	870	0.634
Duration of operation (minutes)	150	180	180	0.088
	N (%)			
Gender				
Male	18 (22.8)	16 (20.2)	16 (20.2)	0.510
Female	9 (11.4)	13 (16.4)	7 (8.9)	
Hypertension	20 (29.4)	25 (36.8)	23 (33.8)	0.756
Diabetes	2 (8) **	9 (36)	14 (56)	0.302
Hyperlipidaemia	2 (7.4) **	14 (51.9)	11 (40.7)	0.313
Hyperparathyroidism	26 (34.2)	28 (36.9)	22 (28.9)	0.692
Principal disease				
Diabetes	1 (1.3)	2 (2.6)	6 (7.6)	0.002 ***
Chronic glomerulonephritis	9 (11.4)	13 (16.5)	4 (5)	
Nephroangiosclerosis	1 (1.3)	6 (7.6)	9 (11.4)	
Polycystic kidney disease	5 (6.3)	4 (5)	1 (1.3)	
Others	11 (13.9)	4 (5)	3 (3.8)	
Renal replacement therapy				
Haemodialysis	21 (26.5)	20 (25.3)	11 (13.9)	0.243
Peritoneal dialysis	5 (6.3)	6 (7.6)	8 (10.1)	
Without dialysis	1 (1.3)	3 (3.8)	4 (5)	
Donor type				
Donation after brain death	25 (31.6)	28 (35.4)	23 (29.1)	0.464
Living-related	2 (2.6)	1 (1.3)	0	
Kidney implantation side				
Right kidney—right side	9 (11.4)	10 (12.6)	11 (13.9)	0.350
Left kidney—right side	12 (15.2)	17 (21.5)	9 (11.4)	
Right kidney—right side	5 (6.3)	1 (1.3)	2 (2.6)	
Left kidney—left side	1 (1.3)	1 (1.3)	1 (1.3)	
Early complications				
Without	12 (15.2)	16 (20.2)	7 (8.9)	0.551
Vascular	4 (5)	1 (1.3)	2 (2.6)	
Urological	0	1 (1.3)	0	
Infective	1 (1.3)	0	0	
Others	3 (3.8)	2 (2.6)	3 (3.8)	
MACE	4 (26.7)	4 (26.7)	7 (46.6)	0.549
One-year graft survival	27 (36.9)	28 (38.4)	18 (24.7)	0.287
One-year patient survival	27 (36)	29 (38.7)	19 (25.3)	0.326

MACE—major adverse cardiovascular events; * PCS Group 1 patients are significantly younger than those from PCS Groups 2 and 3; PCS Group 3 patients are significantly older compared to patients in PCS Groups 1 and 2 (Dunn post hoc analysis); ** Yates correction was applied; *** The number of patients with chronic glomerulonephritis was higher in PCS Groups 1 and 2 compared to PCS Group 3 (post hoc analysis comparison of proportion).

**Table 8:** Renal function indicators one year after kidney transplantation across pelvic calcifications score (PCS) groups. (N = 79).

	PCS Group			
	1 (N = 27)	2 (N = 29)	3 (N = 23)	*p*
	Median (5th–95th Percentiles)			
Urea (mmol/L)	7.5 (4.4–15)	7.65 (4.3–15)	9.4 (6.05–16.29)	0.150
Creatinine (µmol/L)	118 (74–160)	111 (77–191)	109 (73–191)	0.690
MAG-3 clearance (mL/min/1.73 m^2^)	142 (94–245)	162 (57–218)	167 (30–290)	0.896

## Data Availability

The data presented in this study are available on request from the corresponding author. Due to privacy and ethical restrictions, the data contain personal patient information, including names, and therefore are not publicly available.

## Supplementary Materials

The following supporting information can be downloaded at: https://www.mdpi.com/article/10.3390/jcm13206171/s1, Table S1. Demographic and clinical data of patients including pelvic calcifications scores (PCS). Table S2. Correlation Between Pelvic Calcification Scores and Clinical Parameters.
