# Supplementary material for: Impact of Pelvic Calcification Severity on Renal Transplant Outcomes: A Prospective Single-Center Study
Source: J Clin Med. 2024 Oct 16;13(20):6171. doi: 10.3390/jcm13206171 (PMC11508836; doi:10.3390/jcm13206171)
Supplement: Supplementary file 1 [file jcm-13-06171-s001.zip › jcm-3193352-supplementary.pdf]

Table 1. Demographic and clinical data of patients including pelvic calcifications scores (PCS)

Variables		Pelvic calcifications scores (PCS)		
		N	Median (5-95 percentiles)	*P*
Age				
	≤55	36	3 (0-21.8)	<0.001
	>55	43	19 (0-36.7)	
Gender				
	Female	29	12.5 (0-33.1)	0.354
	Male	50	10 (0-36)	
Hypertension				
	Yes	68	13.5 (0-35)	0.017
	No	11	4 (0-18.9)	
Diabetes				
	Yes	25	24 (0-39.5)	<0.001
	No	54	6 (0-25.8)	
Hyperlipidaemia				
	Yes	27	19 (3.85-37.2)	<0.001
	No	52	6 (0-32.9)	
Hyperparathyroidism				
	Yes	76	11.5 (0-35)	0.747
	No	3	18 (0-33)	
Principal disease				
	Diabetes	9	24 (0-33)	0.001*
	Chronic glomerulonephritis	26	10 (0-35.6)	
	Nephroangiosclerosis	16	25 (1.8-41.3)	
	Polycystic kidney disease	10	5.5 (0.36)	
	Others	18	3.5 (0-29.2)	

Renal replacement therapy			
Haemodialysis	52	10.5 (0-36)	0.163
Peritoneal dialysis	19	13 (0-36.6)	
Without dialysis	8	19.5 (5-32)	
Donor origin			
Croatia	44	13.5 (0-36.6)	0.592
Other Eurotransplant countries	35	11 (0-34.5)	
Donor type			
Donation after brain death	76	12.5 (0-35)	0.450
Living-related	3	12	
Kidney implantation side			
Right kidney - left side	30	13 (0-35)	0.170
Left kidney - right side	38	14 (0-30.2)	
Right kidney - right side	8	3 (0-38)	
Left kidney - left side	3	6 (0-44)	
Early complications			
Without	36	11.5 (0-34.1)	0.888
Vascular	10	4.5 (0-35)	
Urological	2	17.5 (11-24)	
Infective	8	16.5 (0-26)	
Others	22	12 (0-39.2)	
MACE			
Yes	15	19 (0-35)	0.128
No	64	10 (0-35.3)	

MACE- major adverse cardiovascular event

*Dunn post hoc analysis patients with diabetes has statistically higher PCS compared to other patients (*P*<0.05); patients with nephroangiosclerosis have statistically higher PCS compared to other patients (*P*<0.05).

Table 2. Correlation Between Pelvic Calcification Scores and Clinical Parameters

Clinical parameters	Pelvic calcification score – Spearman's rang correlation	
	r_s	*P*
Age	0.69	<0.001
Duration of dialysis	0.17	0.123
Duration of operation	0.20	0.079
Length of hospitalization	-0.09	0.445
Graft survival	-0.18	0.116
Patient survival	-0.16	0.147
MACE - major adverse cardiovascular event	0.17	0.129
